# Regulatory T cells in intracerebral hemorrhage: mechanisms of immunomodulation and therapeutic potential

**DOI:** 10.3389/fimmu.2026.1910864

**Published:** 2026-08-03

**Authors:** Jiajie Xia, Xinjie Gao, Shanshan Huang

**Affiliations:** 1 Department of Neurosurgery, Shaoxing Central Hospital, The Central Affiliated Hospital, Shaoxing University, Shaoxing, Zhejiang, China; 2 Department of Neurosurgery of Huashan Hospital, State Key Laboratory of Medical Neurobiology, MOE Frontiers Center for Brain Science, and Institutes of Brain Science, Fudan University, Shanghai, China; 3 Department of Endocrinology, Affiliated Hospital of Jiangnan University, Jiangnan University, Wuxi, Jiangsu, China; 4 National Clinical Research Center for Aging and Medicine, Huashan Hospital, Fudan University, Shanghai, China

**Keywords:** intracerebral hemorrhage, macrophages, microglia, neuroinflammation, regulatory T cells

## Abstract

Intracerebral hemorrhage (ICH) induces profound secondary brain injury, largely driven by dysregulated neuroinflammation, yet effective immunomodulatory therapies remain limited. Regulatory T (Treg) cells, a specialized subset of CD4^+^ T cells essential for immune tolerance and tissue repair, have emerged as critical modulators of post-ICH inflammation and recovery. Following ICH, Treg responses undergo dynamic temporal and spatial changes in the peripheral immune compartment and injured brain, where they may exert neuroprotective effects through multiple mechanisms, including the production of anti-inflammatory cytokines such as IL-10, TGF-β, and IL-35, regulation of microglia/macrophage activation toward reparative phenotypes, promotion of oligodendrocyte precursor cell differentiation, and attenuation of neurotoxic astrogliosis partly through amphiregulin-dependent pathways. Clinical observations suggest that alterations in circulating Treg populations are associated with neurological outcomes after ICH, although these relationships appear to be influenced by disease severity, sampling time, and patient heterogeneity. In experimental models, strategies that enhance Treg number or function, including adoptive Treg transfer, low-dose IL-2, rapamycin, and rCCL17, have been shown to reduce neuroinflammation, limit brain injury, and improve neurological recovery. However, key translational challenges remain, including the optimal therapeutic window, CNS-targeted delivery, antigen specificity, long-term phenotypic stability, and the influence of aging and comorbidities on Treg function. This review synthesizes current evidence regarding Treg dynamics, mechanisms, and therapeutic potential in ICH, with particular attention to emerging approaches such as antigen-specific CAR-Treg cells and gut–brain axis modulation. By integrating mechanistic insights with translational considerations, we propose that Treg-centered immunomodulation represents a promising but still underdeveloped therapeutic strategy for improving recovery after ICH.

## 1 Introduction

Intracerebral hemorrhage (ICH) constitutes the second predominant stroke, representing 10-40% of all incident cases (1–3). Despite considerable survival rates post-initial episode, continued hemorrhage-induced secondary injury causes significant neurological impairment and fatality (4). The low rates of ICH survivors who are functionally independent post-ICH, along with high rates of cognitive decline, result in a significant burden of disease for society and families (5). Current treatment options focus on acute-phase interventions such as ultra-early blood pressure control, anticoagulation reversal, and minimally invasive neurosurgical procedures; bundled approaches combining these measures have shown promise in improving clinical outcomes (6, 7). Despite these efforts, these approaches have failed to adequately prevent secondary brain injury or ensure good long-term functional recovery, leaving patients with persistent disability (8, 9). One major reason may be our incomplete understanding of the underlying pathophysiology, particularly the role of immune-inflammatory responses (10).

The immune system plays a crucial role in the pathophysiology of ICH. Following ictus, robust inflammatory responses are immediately initiated, characterized by the recruitment and differentiation of various immune cells, including microglia, macrophages, astrocytes, and T lymphocytes, alongside the release of pro-inflammatory cytokines and chemokines (11). During the acute phase, these cells predominantly exert detrimental pro-inflammatory functions that contribute to secondary brain injury. However, typically after day 3 (12), they shift toward anti-inflammatory and reparative phenotypes to support tissue recovery. Among these immune populations, CD4^+^ regulatory T (Treg) cells are pivotal for maintaining immune tolerance and promoting an anti-inflammatory microenvironment (13). Treg cells exert immunosuppressive effects through direct cell-to-cell interactions and the secretion of anti-inflammatory cytokines, including IL-10, TGF-β, and IL-35. Consequently, they are increasingly recognized for their vital roles in neuroprotection and neural repair across various neurological disorders. Given their ability to balance immune activation and resolution, targeting Treg cells represents a highly promising therapeutic strategy to modulate post-ICH neuroinflammation, where suppressing excessive inflammation must be balanced with preserving the controlled immune activity essential for debris clearance and tissue repair (14).

Unlike previous reviews that broadly discuss neuroinflammation or immune regulation after ICH, this review specifically focuses on Treg cells as dynamic orchestrators of neuroimmune homeostasis throughout disease progression. We integrate recent advances in Treg biology, spatiotemporal dynamics, intercellular communication, and emerging Treg-targeted therapeutic strategies, while synthesizing evidence from both clinical and preclinical studies. Specifically, we discuss the dynamic changes in Treg populations following ICH, the mechanisms by which Treg cells regulate neuroinflammation and neural repair, current Treg-based therapeutic approaches, and future directions for precision immunotherapy and clinical translation.

## 2 Basic characterization of CD4^+^ regulatory T cells

**Figure 1:**
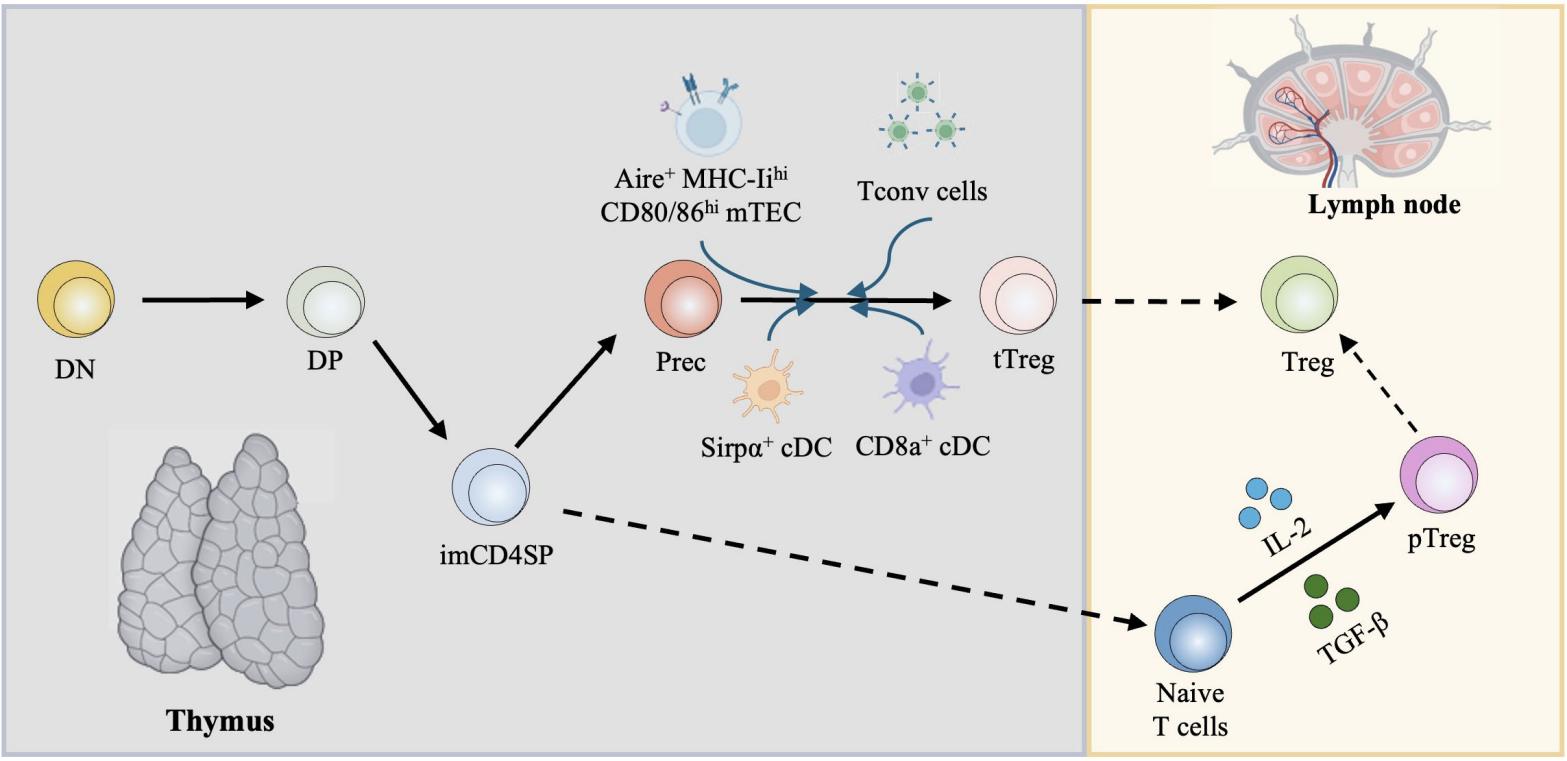
Developmental pathways of tTreg and pTreg cells. Treg cells arise through two distinct developmental pathways. In the thymus, DN thymocytes differentiate into DP thymocytes and subsequently imCD4SP cells. Following TCR recognition of self-peptide–MHC class II complexes and CD28-mediated costimulatory signaling provided by Aire^+^ MHC-II^hi^ CD80/86^hi^ mTECs, Sirpα^+^ cDCs, and CD8α^+^ cDCs, together with IL-2 produced by Tconv cells, Prec mature into tTreg cells. Mature tTregs subsequently exit the thymus and populate peripheral lymphoid organs. In the periphery, naïve CD4^+^ T cells can also differentiate into pTreg cells under the combined influence of TGF-β and IL-2. Both tTreg and pTreg populations contribute to the maintenance of immune tolerance and immune homeostasis. DN, double negative T cells; DP, double positive T cells; imCD4SP, immature CD4 single positive T cells; Prec, Treg precursor cells; tTreg, thymic Treg cells; pTreg, peripheral Treg cells; Tconv, conventional T cells; cDC, conventional dendritic cell; mTEC, medullary thymic epithelial cell.

Treg cells are a subset of T cells, accounting for only 3-5% of all T lymphocytes in peripheral blood (15). The combined expression of CD4, CD25, and the specific transcription factor Forkhead Box P3 (Foxp3) has been used to identify Treg cells in rodents (16). Treg cells can be generally divided into two types. The first type is directly derived from the thymus, known as thymus-derived Treg (tTreg) or naturally occurring Treg (nTreg), which is the result of high-affinity interaction with the self-peptide presented via the major histocompatibility complex (MHC) during the development of T cells in the thymus (13, 17). The other type is peripheral-derived Treg (pTreg) or inducible Treg cells (iTreg) (18), such as Th3, Tr1, CD8^+^ Treg, and natural killer Treg cells. pTreg cells originate from peripheral naive CD4^+^ T cells, and their differentiation process is determined by antigen stimulation and related cytokines, such as interleukin (IL)-2 and transforming growth factor (TGF)-β (**Figure 1**) (19).

FoxP3 is an X-linked transcription factor that plays a crucial role in the differentiation, maintenance, and function of Treg cells (19). Its expression level determines the immunosuppressive properties of Treg cells (20), and nearly all inhibitory Treg cells express FoxP3 (21). The precursor of Treg cells receives signals from T cell receptor (TCR) and CD28 ligands, inducing transcription factors such as nuclear factor kappa-B (NF-κB), activator protein (AP)-1, and nuclear factor of activated T cells (NFAT), which initiate the transcription of FoxP3. Foxp3 protein mainly regulates TCR gene expression through the acute myeloid leukemia 1/Runt-related transcription factor 1 (AML1/Runx1) signaling pathway and produces negative feedback on nuclear factors such as NFAT, which further regulates Foxp3 gene expression and Treg cell differentiation (22). Mutations in the FoxP3 gene lead to its dysfunction, resulting in severe diseases such as IPEX syndrome (23–25). In the context of brain hemorrhage, FoxP3 may modulate the immunosuppressive functions of Treg cells, influencing inflammatory responses and neural repair, thus offering potential therapeutic targets.

CD25 is a component of the high-affinity IL-2 receptor, which is expressed on the surface of almost all Treg cells and is essential for the survival of Treg cells (26). IL-2 can stabilize the expression of Foxp3 and regulate the production of surface molecules such as cytotoxic T lymphocyte-associated protein (CTLA)-4 and tumor necrosis factor (TNF) receptor on the surface of Treg cells by inducing the production of Foxp3 mRNA, thereby maintaining the stability of the cells.

Treg cells are primarily localized in the blood and lymphoid tissues, but they also migrate into non-lymphoid tissues like skin, adipose tissue, muscle tissue, brain, lung, and colon under homeostatic conditions. However, during inflammatory responses, Treg cells accumulate in these tissues. The brain was traditionally considered an immune-privileged organ, with the assumption that lymphocytes were excluded from the healthy brain. However, some immunological experiments have challenged this notion (27–29). These experiments have demonstrated that prior exposure to graft tissue in the periphery can facilitate rapid rejection of the graft in the brain. This finding indicates that antigen-experienced lymphocytes can enter and function within the brain, challenging the classical view of brain immunoprivilege. The brain Treg cell population is dynamic over the life course of mice, with the first detection of these cells occurring around the time of birth and an increase in the population observed with both age and environmental enrichment (30). Based on their prominent expression of Helios and Neuropilin 1, most brain Treg cells are conventionally identified as being of thymic origin, a lineage further supported by the restricted diversity of their T cell receptor repertoire following ischemic stroke (30, 31). Multiple potential migration routes are allowing Treg cells to enter brain parenchyma (1). Blood-brain barrier (BBB): Treg cells would need to extravasate and reach the perivascular space and cross the glia limitans to the brain parenchyma (32). This is likely the primary route underlying Treg cell infiltration into the peri-hematomal tissue after intracerebral hemorrhage (33) (2). Blood–cerebrospinal fluid (CSF) barrier: Treg cells would need to translocate the choroid plexus epithelium and cross the ependyma into the brain parenchyma (34) (3). Brain surface barrier: an entry route for Treg cells from the skull marrow to the meningeal compartments (35) (4). Brain–cribriform plate barrier: lymphatics near the cribriform plate may have direct access to CSF through gaps in the arachnoid barrier (36) (5). Extravasating directly from parenchymal blood vessels into the injured brain (37). However, these pathways have not been fully validated.

Treg cells exhibit both shared and tissue-specific characteristics across different organs (38). In the brain, Treg cells retain canonical Treg markers, including CD69, CTLA-4, PD-1, GITR, CD103, Helios, KLRG1, and ST2, while acquiring a distinct transcriptional program characterized by the expression of central nervous system (CNS)-associated genes such as *Htr7*, *Npy*, *Avpr1a*, and *Penk* (31, 39, 40). Their accumulation within the CNS is orchestrated by CCL1- and CCL20-mediated recruitment together with IL-2-, IL-33-, serotonin-, and TCR-dependent expansion and maintenance. Notably, the serotonin receptor 5-HT7, encoded by *Htr7*, promotes the expansion of brain Treg cells and contributes to neurological recovery following CNS injury (31, 41). In addition, brain Treg cells express abundant immunoregulatory and tissue-repair molecules, including IL-10, AREG, and PPARγ, supporting their specialized functions in maintaining CNS immune homeostasis and tissue repair (31).

Beyond their unique phenotype, brain Treg cells perform multiple neuroprotective functions that are essential for maintaining CNS homeostasis and facilitating tissue repair. They secrete OPN, which suppresses the excessive activation of microglia and infiltrating immune cells while promoting the polarization of glial cells toward a reparative phenotype, thereby limiting neuroinflammation (42). Treg cells also protect neurons from neurotoxicity and enhance neuronal survival (43). Furthermore, they promote remyelination through the secretion of CCN3, which stimulates the differentiation of oligodendrocyte precursor cells into mature oligodendrocytes (44). In addition, Treg cells facilitate neurogenesis by promoting the proliferation of neural stem cells (45). Emerging evidence further suggests that brain Treg cells contribute to systemic metabolic homeostasis by regulating energy balance and glucose metabolism through interactions with the hypothalamus (46). Collectively, these findings demonstrate that brain Treg cells not only suppress excessive neuroinflammation but also actively coordinate neuronal protection, remyelination, neuroregeneration, and metabolic regulation. In the context of ICH, these tissue-adapted Treg cells are therefore uniquely positioned to orchestrate the resolution of neuroinflammation, promote tissue repair, and improve neurological recovery.

## 3 Changes in Treg cells after ICH

### 3.1 Clinical evidence on Treg dynamics in ICH

Previous studies have consistently reported an elevated percentage of peripheral Treg cells during the acute and subacute phases of ICH (47, 48). However, when correlating these circulating Treg dynamics with clinical severity, divergent findings have been reported. As summarized in **Table 1**, Shi et al. demonstrated that patients with severe neurological deficits, defined by a National Institutes of Health Stroke Scale (NIHSS) score > 20, exhibited a higher frequency of circulating Treg cells at days 3 and 7 after ICH (47), whereas Jiang et al. reported a lower proportion of circulating Treg cells in patients with severe impairment, defined by a Glasgow Coma Scale (GCS) score ≤ 7, within 30 hours after onset (48).

**Table 1:** Summary of clinical studies evaluating peripheral Treg responses following ICH.

Study characteristic	Shi et al.	Jiang et al.
Patient Cohort (n) & Timing	Day 3: n = 68 (38 M/30 F) Day 7: n = 65 (36 M/29 F)	Within 30 h: n = 35 (19 M/16 F)
Sample Source	Peripheral blood	Peripheral blood
Gating Strategy	CD4+ CD25high CD127low/− FoxP3+	CD4+ CD25+ FoxP3+
Treg quantification	Percentage of CD4+ T cells	Percentage of total lymphocytes
Major Findings	Peripheral Treg frequency increased	Peripheral Treg frequency increased
Correlation with Severity	At days 3 and 7, Treg percentages were significantly higher in the severe group (NIHSS > 20) than in the mild-to-moderate group.	Within 30 h, Treg percentages were significantly lower in the severe group (GCS ≤ 7) than in the milder group.

The discrepancies among these studies are likely attributable to methodological and clinical heterogeneity rather than true biological inconsistency. First, circulating Treg cells undergo dynamic changes throughout the hyperacute, acute, and subacute phases of ICH, and different sampling time points may therefore capture distinct stages of the immune response. Second, disease severity was assessed using different clinical scales. The NIHSS primarily evaluates neurological deficits, whereas the GCS assesses the level of consciousness, making direct comparisons between studies challenging. Third, differences in flow cytometric gating strategies may affect Treg quantification, as the inclusion of CD127 improves discrimination of bona fide Treg cells from activated conventional CD4^+^ T cells. In addition, the two studies quantified Treg cells using different denominators, which may further contribute to inconsistent findings. Finally, host-related factors, particularly age and comorbidities, may also influence circulating Treg responses. Age has been associated with reduced peripheral Treg frequencies and is itself an independent predictor of ICH severity (48, 49), suggesting that age-related immune alterations may confound the relationship between Treg dynamics and clinical outcomes. Therefore, future clinical studies should adopt standardized sampling schedules, unified Treg phenotyping strategies, and consistent clinical assessment criteria while accounting for patient-related confounding factors to better define the clinical significance of Treg cells in ICH.

### 3.2 Preclinical evidence from animal models

Previous studies have shown that no difference in T lymphocytes and Treg cells in the peripheral blood was found between ICH and sham rats at day 3 after ICH onset (50, 51). However, another study shows that Treg cell ratios were significantly lower in the model groups in the peripheral blood compared with the sham groups at day 5 after ICH onset (52).

In contrast to the inconsistent changes observed in peripheral blood, most experimental studies have demonstrated a progressive accumulation of Treg cells within the injured brain after ICH. Although brain Treg cells are scarce under homeostatic conditions, their population can expand to several hundred per 10^6^ brain cells following ischemic stroke (42). In the context of ICH, the absolute numbers of brain-infiltrating T lymphocytes significantly increase compared to sham-operated controls, with CD4^+^ T cells emerging as the predominant leukocyte subpopulation (50, 53). Naive CD4^+^ T cells were activated and differentiated into different phenotypes, including T-helper 1, T-helper 2, T-helper 17, and Treg cells (54). The number of Treg cells within ipsilateral peri-hematoma tissue increased from the 1st day, and this trend was maintained until 7 days after ICH (33, 50, 55). However, another study showed that they had similar numbers of Treg cells in the ipsilateral hemisphere in the control and ICH groups at day 3 after ICH onset (51).

Collectively, available animal studies support dynamic regulation and progressive accumulation of Treg cells within the injured brain after ICH, although the reported kinetics vary across studies. These discrepancies are likely attributable to differences in experimental models, ICH induction methods, sampling time points, and Treg identification strategies. Standardized experimental protocols combined with spatially resolved immune profiling will be important for defining the temporal and functional characteristics of Treg responses after ICH.

## 4 Mechanisms of Treg-mediated immunomodulation and repair after ICH

Secondary injuries following ICH evolve over days to weeks, driven by inflammatory, toxic, and oxidative pathways that intensify perihematomal damage (56–60). BBB breakdown and upregulation of cell adhesion molecules in the perihematomal region cause the recruitment of systemic leukocytes including innate immune cells (natural killer cells, monocytes, and neutrophils), and the adaptive immune system including T cells (53).

**Figure 2:**
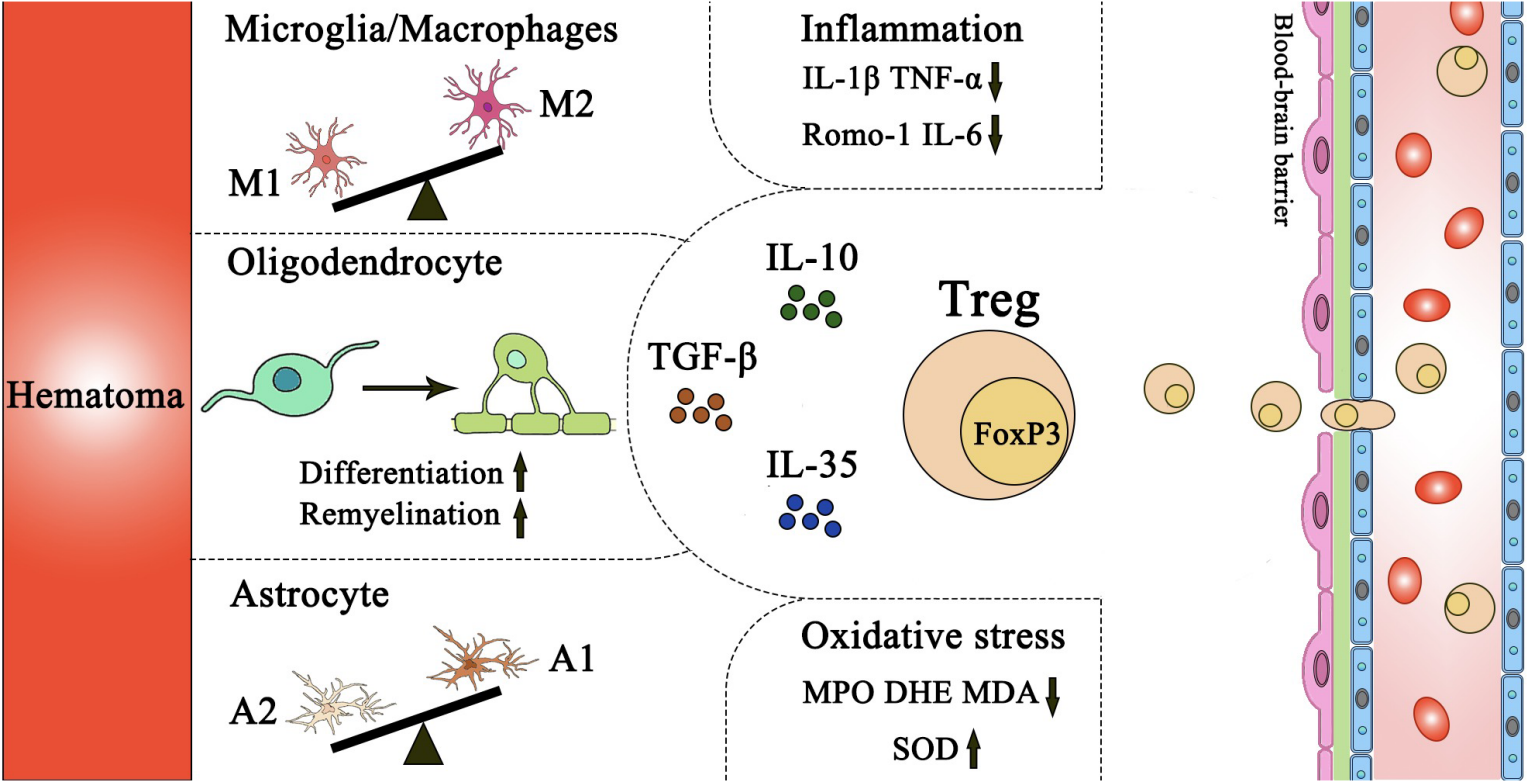
Impacts of Treg cells on brain parenchyma after ICH. Treg cells extravasate and reach the perivascular space, subsequently crossing the glia limitans to enter the perihematomal region. Treg cells effectively reduce the levels of pro-inflammatory cytokines and oxidative stress. Treg cells secrete cytokines, including IL-10, TGF-β, and IL-35, to suppress immune responses. Additionally, Treg cells facilitate a shift of microglia/macrophages from inflammatory toward reparative activation states. They drive the differentiation of oligodendrocyte precursor cells into oligodendrocytes, accelerating both developmental myelination and remyelination processes. Furthermore, Treg cells suppress neurotoxic A1-like astrogliosis by producing AREG, thereby promoting neurological recovery.

A large number of T cells infiltrating the hemorrhagic hemisphere began to accumulate on the first day and peaked on the fifth day after ICH (61–64). These T cells in the hemorrhagic brain originate from both the hematoma and the peripheral blood, with the majority deriving from systemic circulation (53). Treg cells, as cells of thymic origin, activated in lymphoid organs, need to traffic to the brain via the blood. Treg cells cross to the brain parenchyma to exert their functions of immunoregulation and tissue repair by a variety of cellular and molecular mechanisms (**Figure 2**).

### 4.1 Modulation of neuroinflammation via cytokine secretion

Neuroinflammation has been recognized as a critical component of ICH pathophysiology. The inflammation and oxidative stress are triggered by molecules released from damaged or dead cells and hemoglobin, heme, and iron released after red blood cell lysis (65). Neuroinflammation can promote to clear away dead cells and debris, promote neural repair, and support neurogenesis. However, it can also contribute to BBB disruption, edema formation, and neuronal death, exacerbate brain damage leading to secondary injury, impeding tissue repair and recovery, and contribute to post-stroke complications, such as motor and cognitive impairment (66).

The cytokines secreted by Treg cells, including IL-10, TGF-β, and IL-35, are known to suppress immune responses involving effector T lymphocytes and other immune cells.

IL-10 secreted by Treg cells alleviates neural injury and promotes neurogenesis (67–69). Intranasal administration or liposome delivery of IL-10 accelerated hematoma clearance, regulated brain inflammation, inhibited glial activation, and improved neurologic function (70, 71). IL-10 activates the phosphorylation of ERK and STAT3 and regulates the subventricular zone (SVZ) adult neurogenesis (72).

TGF-β promotes activation of anti-inflammatory microglia/macrophages, reduces the inflammatory response, and promotes functional recovery after ICH (73, 74). TGF-β enhances adult neurogenesis in the hippocampus and the SVZ (75, 76). Treg cell depletion decreased the expression of TGF-β, aggravated early brain injury, and deteriorated neurological deficits after ICH (33). A significant increase in IL-10 and TGF-β in the peripheral blood is observed after ICH (47, 48). Patients with a favorable outcome had significantly higher levels of IL-10 in the peripheral blood, and higher levels of IL-10 and TGF-β in the hematoma compared to patients with an unfavorable outcome (48).

IL-35, an anti-inflammatory and neuroprotective cytokine, is preferentially secreted by Treg cells and plays a potent immunosuppressive role in various diseases (77–79). This cytokine contributes to T cell exhaustion and inhibits the proliferation of effector T cells (80). CD4^+^ T cells, which are recruited into the brain, worsen ICH injury by promoting pro-inflammatory and pro-apoptotic actions (81). IL-35 induces microglial polarization toward the M2 phenotype by inhibiting JNK signaling and activating JAK2/STAT6 signaling (82). This modulation of microglial phenotype is believed to contribute to the neuroprotective effects of IL-35.

Treg cell depletion increased inflammatory cytokines such as IL-1β, TNF-α, and Romo-1, and increased oxidative stress markers such as Myeloperoxidase (MPO) and dihydroethidium (DHE) (33). Transplanted Treg cells reduced the levels of pro-inflammatory cytokines (IL-1β, IL-6, TNF-α) and oxidative stress markers (such as malondialdehyde) while increasing the activity of the antioxidant enzymes (such as superoxide dismutase) and decreasing the number of inflammatory and apoptotic cells after ICH (83).

### 4.2 Regulation of microglia/macrophage activation states

Beyond systemic immunosuppression, Treg cells directly interact with glial cells to shape the post-ICH microenvironment. As the key active immune defender intrinsic to the CNS, microglia/macrophages are recognized as the first non-neuronal cells to respond to ICH (84). Microglial activation plays a crucial role in the pathology and progression of ICH. Activated microglia/macrophages can develop M1-like (proinflammatory) or M2-like (anti-inflammatory) phenotypes in response to ICH. Modulating the microglial phenotype has the potential to not only expedite the absorption of hematoma and edema but also enhance white matter integrity, foster brain repair, and aid in functional recovery (85, 86).

Treg cells have been shown to inhibit microglia/macrophage activation through the JNK/ERK signaling pathway and NF-κB inactivation, subsequently suppressing the expression of TNF-α, IL-1β, and MMP-2 (87). Concurrently, Treg cells promote M2-like polarization through the TGFβ/TGFβR/Smad2/3 (33) and PI3K/Akt/mTOR (55), pathways, and their depletion significantly shifts the balance toward M1-like states in ICH mice (33). Beyond linear polarization, Treg cells directly fine-tune microglial phagocytic and efferocytotic machinery to facilitate tissue remodeling. Mechanistically, Treg-derived OPN can suppress microglial CD74 expression, establishing a critical OPN-CD74 axis that enhances debris clearance and synaptic engulfment (88). Additionally, brain-infiltrating Treg cells can directly amplify microglial phagocytic capacity and dampen chronic neuroinflammation in an IL-10/IL-10R-dependent manner (89).

Furthermore, this crosstalk represents a reciprocal homeostatic and metabolic feedback loop. During tissue clearance, myelin-phagocytosing microglia upregulate MHC class II to sustain local Treg survival and clonal expansion (90). In turn, licensed Treg cells mitigate microglial inflammation via CTLA-4 and upregulate the Abcg1 receptor on microglia, optimizing lipid load management and preventing toxic lipid droplet formation to preserve white matter. This interplay is further reinforced by direct immune checkpoints, where Tregs utilize the PD-L1/PD-1 cascade to contact microglia, restoring physiological microglial morphology and arborization (91).

### 4.3 Facilitation of OPC differentiation and remyelination

After ICH, white matter injury may also lead to severe sensorimotor dysfunction, neurobehavioral impairment, and cognitive disorders (92). Oligodendrocyte precursor cells (OPCs) help to repair the myelin sheath and restore white matter integrity by differentiating into new mature oligodendrocytes that produce myelin. Treg cells promote OPC differentiation into oligodendrocytes, thereby accelerating developmental myelination and remyelination (93). In demyelinating diseases, this acceleration of oligodendrocyte differentiation and (re)myelination is mediated by CCN3, produced by Treg cells (44). Additionally, Treg cells enhance myelin production in ischemic stroke (94), and depletion of Treg cells impairs oligodendrocyte differentiation and remyelination *in vivo*. Notably, while young Treg cells effectively support remyelination, their regenerative capacity declines with aging due to intrinsic functional impairments (95), suggesting that rejuvenating aged Treg cells or harnessing young-like Treg properties could be a promising therapeutic strategy for enhancing recovery after brain injury.

### 4.4 Suppression of neurotoxic astrogliosis

Astrocytes maintain CNS homeostasis; the homeostatic function of astroglia is linked to their neuroprotective capabilities (96). Neuroinflammation and ischemia resulted in two different phenotypes of reactive astrocytes, termed A1 and A2 astrocytes (97). The A1 neurotoxic astrocytes lose normal astrocytic functions and induce the death of neurons, oligodendrocytes, and OPCs. ICH could strongly induce A1 neurotoxic astrocytes (98). Treg cells suppress neurotoxic astrogliosis by producing AREG and promote neurological recovery (31). Recent studies in other CNS injury models further indicate that Treg cells regulate astrocyte function through multiple mechanisms, including IL-10-mediated inhibition of reactive astrogliosis, suppression of STING signaling to enhance astrocyte-neuron lactate shuttle, and modulation of the HIF-1α/Hmox1 axis to limit astrocytic ferroptosis, collectively facilitating tissue repair and neurological recovery (99–101). Although these findings highlight astrocytes as important targets of Treg-mediated neuroprotection, whether these mechanisms operate in ICH and how they are temporally coordinated remain largely unknown.

The majority of studies of Treg cells in anti-inflammatory response and neural repair process focus on Treg cell function in acute phases of ICH and at relatively short timeframes after ICH. These phases typically exhibit enhanced permeability of the BBB, and glial scars haven’t formed, which facilitates the recruitment of Treg cells to the perihematomal region. Extensive experiments are still necessary to validate the importance of Treg cells in post-ICH brain recovery, especially when BBB function is restored, and glial scars may have formed. However, extensive research has been conducted on the mechanisms through which Treg cells impact the brain. The utilization of single-cell sequencing technology has facilitated the exploration of immune cell populations and their interactions with other cells, revealing that cell-cell crosstalk may play a crucial role in enabling the limited Treg cell population to exert profound effects on the injured brain. Engineered Treg cells with enhanced protective mechanisms are currently being developed and anticipated for adoptive cell therapy as a substitute for broad-spectrum unspecific immunotherapies.

## 5 Treg cells for ICH treatment

### 5.1 The potential of Treg therapy in neurological disorders

As key factors and mechanisms underlying Treg biology continue to be elucidated, Treg therapy is emerging as a promising therapeutic strategy. Treg therapy’s implications have spanned a multitude of diseases including graft versus host disease, cancer, diabetes, ulcerative colitis, Crohn’s disease, IPEX syndrome, aplastic anemia (AA), systemic lupus erythematosus (SLE, lupus), acute respiratory distress syndrome (ARDS), alopecia areata, end-stage liver disease and neurological disorders like Multiple Sclerosis (MS), amyotrophic lateral sclerosis (ALS) and Alzheimer’s disease (AD) in **Table 2**, as documented on ClinicalTrials.gov. Treg therapy involves either the adoptive transfer of ex vivo expanded By expanding Treg cells, researchers can explore their immunoregulatory potential across various disease models. In neurological disorders, Treg cells have demonstrated the ability to reduce neuroinflammation and protect neurons. Based on these findings, Treg therapy may offer a novel therapeutic approach for neuroprotection and recovery following ICH.

**Table 2:** Treg therapy in neurological disorders.

Study phase	Condition/disease	Intervention/treatment	ClinicalTrials.gov ID
Phase 2a	Amyotrophic Lateral Sclerosis	Autologous Treg cells + IL-2	NCT04055623
Phase 1	Guillain-Barré Syndrome	CK0801 (Cord blood-derived Treg cells)	NCT03773328
Phase 1	Multiple Sclerosis	ABA-101 (a TCR engineered, autologous Treg therapy)	NCT06566261
Phase 2	Alzheimer’s Disease	Treg cells	NCT03865017
Phase 2	Bipolar disorder	Low dose IL-2	NCT04133233
Phase 1	Amyotrophic Lateral Sclerosis	CK0803 (neurotrophic allogenic Treg cells)	NCT05695521
Phase 1	Alzheimer’s Disease	VT301 (Treg cells)	NCT05016427
Phase 2	Alzheimer’s Disease	Low dose IL-2	NCT06096090
Phase 2	Alzheimer’s Disease	Low dose IL-2	NCT05468073
Phase 1	Alzheimer’s Disease	Low dose IL-2	NCT05821153
Phase 1	Amyotrophic Lateral Sclerosis, Alzheimer’s Disease, Multiple System Atrophy	Treg cells	NCT06671236
Phase 1	Amyotrophic Lateral Sclerosis	Treg cells + IL-2	NCT03241784
Phase 2	Multiple Sclerosis	Low dose IL-2	NCT02424396

Notably, multiple phase I/II trials are currently evaluating Treg-targeted interventions in AD, ALS, and MS, demonstrating growing interest in harnessing regulatory immunity for neuroprotection. Although no trial has yet focused specifically on ICH, the success of low-dose IL-2 and CAR-Treg platforms in other conditions provides strong rationale for extending these approaches to hemorrhagic stroke.

### 5.2 Treg expansion ex vivo

In preclinical studies, adoptive Treg therapy is the most common approach to expanding Treg cells by transfusing Treg cells isolated from animals’ inguinal, axillary lymph nodes, and spleens (83, 87). The strategies for Treg expansion ex vivo include polyclonal and antigen-specific Treg cells. The most used approach is the expansion of polyclonal Treg cells purified (magnetic bead-isolated or flow-sorted) from circulating Treg cells. To ensure highly selective Treg cell expression, rapamycin is often used to prevent the proliferation of T effector cells (102, 103). However, this suppression could affect Treg cells, which could result in the limited rate of the expansion of Treg cells. A part of the Treg cells rapidly disappeared within a few days of the transfer (104), possibly due to their high expression of granzymes and proneness to undergo apoptosis (105). In addition, Treg cells can be re-programmed to T helper (Th)17 cells in the presence of TGF-β and IL-6 (106). To address some of these limitations, researchers have developed antigen-specific Treg cells, including chimeric antigen receptor (CAR) Treg cells, to increase the survival, numbers, purity, antigen specificity, and functional stability.

The enhanced trafficking of antigen-specific Treg cells towards target tissues presumably permits the utilization of fewer Treg cells without the risk of broad immunosuppression in comparison to polyclonal strategies, thus optimizing the clinical application of Treg-based immunotherapy (107). Donor antigen-alloreactive Treg cells can be stimulated specifically by antigen-presenting cells (peripheral blood mononuclear cells, B cells, or dendritic cells) from the transplant donor, and used mainly in transplantation. These antigen-specific Treg cells can reduce off-target immunosuppressive activity, but their yield is comparatively low after expansion. Engineering antigen-specific Treg cells can be produced through genetic insertion of synthetic receptors such as TCR (108), CAR (109), or B cell antibody receptor (BAR) (110, 111). The translation of TCR-engineered Treg cells into clinical practice is somewhat hindered by their MHC restriction and the necessity to isolate and identify antigen-specific and disease-relevant TCRs. However, CAR Treg cells and BAR Treg cells do not exhibit MHC restriction, which may facilitate their clinical application. Numerous preclinical studies have demonstrated that antigen-specific Treg cells may be both safer and more efficient than unselected polyclonal Treg cells for adoptive cell therapy (112–114). Furthermore, antigen-specific Treg cells primarily localize at the site of antigen presentation, thereby minimizing the risk of generalized immunosuppression. For neurological disorders such as intracerebral hemorrhage, ex vivo expansion of antigen-specific or polyclonal Treg cells offers a promising therapeutic avenue, and optimizing their delivery and persistence in the brain could enhance their efficacy in promoting recovery.

### 5.3 Enhancement of Treg cell number and function *in vivo*

Direct administration intravenously of Treg cells is not feasible for ICH owing to the off-target activity or BBB. Administration of numerous agents has been shown to enhance Treg cells in ICH models. Their effects are documented in **Table 3**.

**Table 3:** Strategies to increase Treg cells frequency and function for ICH treatment.

ICH model	Treatment	Animal	Observed effect(s)	Ref
Autologous blood	Minocycline	Piglets	Improved CD4^+^ T cell differentiation to Treg cells; reduced neuroinflammation and white matter injury	(54)
Autologous blood	SIRPα-v Exos	C57BL/6 mice	Increased Treg cells in the peri-hemorrhagic region; coordinately modulated the biofunction of microglia/macrophages	(55)
Autologous blood	PD-L1	C57BL/6 mice	Increased the numbers of Treg cells in the brain; ameliorated the inflammatory milieu; decreased cell death; enhanced BBB integrity; attenuated neurological deficits	(115)
Autologous blood	Liangxue Tongyu prescription	Sprague-Dawley (SD) rats	Maintained the balance of Th17/Treg Cell in the peripheral blood; ameliorated neurological deficits, brain edema, BBB permeability, and neuroinflammation	(52)
Collagenase type IV	Rapamycin	SD rats	Increased the number of Treg cells in peripheral blood and brain; increased the levels of IL-10 and TGF-β, reduced the level of interferon-gamma (IFN-γ) both in peripheral blood and brain; improved the neurobehavioral deficit	(51)
Autologous blood	rCCL17	CD-1 mice	Increased Treg cells in the brain; promoted M2 microglia/macrophages polarization; attenuated early brain injury	(33)
Collagenase type IV	Neural stem cells	SD rats	Increased Treg cells in the brain; protected brain injury	(50)

The mTOR pathway tightly regulates the suppressive function of Treg cells, and any alteration in its activity, whether through inhibition or overactivation, can impact both the stability and functionality of Treg cells (116). PD-L1 affects the phenotype shift of CD4^+^ T cells by inhibiting the mTOR pathway, leading to an increase in the percentage of Treg cells (115), while rapamycin also increases the number of Treg cells by inhibiting the mTOR pathway, enhancing their suppression function and promote neurologic functional recovery (51). Notably, Treg cells exhibit elevated basal mTORC1 activity compared to conventional T cells, which is essential for their suppressive function and metabolic fitness, particularly through the regulation of cholesterol metabolism (117). The therapeutic efficacy of rapamycin in boosting Treg function highlights a paradox: while mTOR signaling is crucial for Treg stability, its pharmacological inhibition can selectively expand functional Treg populations. This delicate balance underscores the importance of context-specific modulation of the mTOR pathway for optimal Treg-based interventions in diseases like ICH.

Several other signaling molecules have the potential to promote the expansion and/or function of certain Treg subsets more selectively *in vivo*, like the target of rCCL17 (CCR4) (33, 118) and minocycline (Notch1) (54). Previous studies have demonstrated that activation of the CCL17/CCR4 axis facilitates the recruitment of Treg cells *in vivo* (119, 120). CCL17 specifically binds to CCR4, which is expressed in a subpopulation of Treg cells (121). Administration of exogenous recombinant CCL17 (rCCL17) enhances the chemotaxis of Treg cells into the peri-hematomal area following blood-brain barrier disruption after ICH (33). Previous reports have established the significance of the Notch signaling pathway in regulating and directing the differentiation of diverse CD4+ T cell subsets (122). The Notch ligand, Jagged-1, has been shown to facilitate the expansion of Treg cells (123). Nevertheless, some studies have contradicted this notion, indicating that Notch signaling can attenuate Treg function (124–126). In the ICH model, the treatment with minocycline favored the preferential differentiation of CD4^+^ T cells towards a Th2 and Treg phenotype (54). Whole transcriptome sequencing showed an increased expression of T cell activation and differentiation-related genes after ICH, including the Notch signaling pathway (54). Previous reports have demonstrated the role of the Notch signaling pathway in regulating CD4^+^ T cell differentiation (122, 125, 127).

There are several basic modalities of Treg cell therapy *in vivo* that are currently receiving the most attention, one of which is IL-2 therapy. IL-2 is essential for Treg survival and function. IL-2Rβ−/− mice do not produce Treg cells in the thymus; however, transgenic thymic transgenic expression of wild-type IL-2Rβ in these mice reconstitutes normal Treg development (128). IL-2 signaling through the STAT5 pathway is pivotal to Treg cell development (129). The elevated expression of IL-2Rα on Treg cells confers a competitive edge in binding IL-2, even at subphysiological concentrations (130). Targeted modulation of Treg cells through low-dose IL-2 therapy not only increases their numbers but also increases their immunosuppressive capabilities. Numerous studies have shown that low-dose IL-2 therapy significantly improves clinical symptoms in patients or preclinical mouse models of Alzheimer’s disease (131), amyotrophic lateral sclerosis (132), migraine (133), depression (134), and various other neurological conditions (135–137). This makes IL-2 therapy an exceptionally promising therapeutic strategy for a wide range of neuroinflammation conditions. However, its application in the context of ICH remains underexplored.

Beyond direct pharmacological or cytokine-based interventions, emerging evidence highlights the role of systemic physiological networks—particularly the gut-brain axis—in modulating neuroinflammation through immune regulation. The gut microbiome has a modulatory role in directing host systemic immune responses and can enhance Treg generation *in vivo* as a strategy to enhance organ tolerance. Recent studies (52, 138) have shown that the intestinal fora and its metabolites can induce Foxp3 expression via GPR43, regulate the Th17/Treg ratio, and ameliorate neuroinflammation in ICH rats. Strategies involving stem cell-based approaches or nanovesicles to enhance Treg cell migration are also being explored (50, 55).

Treg therapy offers a diverse range of immunological approaches for the potential clinical management of ICH. However, several challenges, including determining the optimal intervention window and achieving precise targeting and delivery of Treg cells to the injured site within the brain, must be addressed before these therapies can be translated into clinical practice. In summary, while Treg therapy offers promising potential as a treatment for ICH, further research is crucial to elucidate their mechanisms of action, refine delivery strategies, and establish their safety and efficacy in human clinical trials.

### 5.4 Challenges and clinical limitations of Treg-targeted therapies

Despite encouraging preclinical results, several barriers currently limit the clinical translation of Treg-based therapies for ICH in **Table 4**. Adoptive Treg transfer is constrained by inefficient CNS homing, limited *in vivo* persistence, and the potential risk of systemic immunosuppression. Although antigen-specific Tregs and CAR-Tregs may improve tissue selectivity, disease-relevant target antigens in ICH remain poorly defined, and individualized cell manufacturing remains technically demanding. Pharmacological approaches, including low-dose IL-2 and rapamycin, offer greater clinical accessibility but lack sufficient Treg specificity and require repeated or prolonged administration, raising concerns regarding off-target immune modulation and infection risk. Modulation of the gut–brain axis represents a non-invasive alternative; however, substantial inter-individual variability in microbiota composition and the absence of standardized therapeutic protocols currently limit reproducibility and clinical translation. Collectively, overcoming these challenges will require improvements in targeted delivery, long-term stability, antigen specificity, and rigorous clinical validation before Treg-targeted therapies can be widely implemented in ICH.

**Table 4:** Advantages and limitations of potential Treg-targeted therapeutic strategies for ICH.

Strategy	Delivery	Safety	Antigen specificity	Durability	Clinical feasibility
Polyclonal Treg cells	Intravenous	Risk of systemic immunosuppression; potential Treg instability	Low	Limited persistence; requires repeated dosing	Technically feasible but labor-intensive and costly (139, 140)
Antigen-specific Treg cells	Intravenous	Improved safety with reduced off-target immunosuppression; potential gene-engineering risks	High	Greater persistence than polyclonal Tregs	Limited by manufacturing complexity, antigen identification, and regulatory challenges (141, 142)
Low-dose IL-2	Intravenous	Generally well tolerated; excessive dosing may activate effector T/NK cells	Low	Requires repeated administration because of short half-life	Approved for other indications;limited by a narrow therapeutic window (143, 144)
Rapamycin	Oral or Intravenous	Dose-dependent immunosuppression; long-term use carries high risk of post-stroke pneumonia	Low	Sustained only during treatment	Approved for other indications; non-specific mTOR pathway modulation (145, 146)
Gut-brain axis modulation	Oral	Generally favorable safety profile	Low (indirect)	Highly variable; dependent on microbiota stability	Clinically attractive but lacks standardized protocols (14, 147)

## 6 Conclusion

Treg cells have emerged as pivotal regulators of immune homeostasis and neural repair following ICH. Through multifaceted mechanisms, including the suppression of pro-inflammatory responses, promotion of microglial M2 polarization, inhibition of neurotoxic astrogliosis, and facilitation of oligodendrocyte differentiation, Treg cells orchestrate a coordinated transition from destructive inflammation to tissue restoration. Notably, their functional role extends beyond the acute phase. Early Treg activation mitigates secondary brain injury by dampening excessive neuroinflammation, whereas accumulating evidence indicates that Treg cells continue to promote neurological recovery during the subacute phase by enhancing white matter repair and facilitating the resolution of glial activation.

Preclinical studies consistently demonstrate that augmenting Treg numbers or function through adoptive transfer, low-dose IL-2, or pharmacological agents such as rapamycin and rCCL17 improves neurological outcomes in ICH models. However, the therapeutic application of Treg-based strategies faces several challenges. First, the dynamic nature of post-ICH immunity necessitates precise timing of intervention, as immunomodulation is protective during the early stage, whereas excessive or inappropriate suppression may interfere with endogenous clearance and tissue remodeling. Second, systemic delivery carries the risk of off-target immunosuppression, highlighting the need for targeted approaches, including antigen-specific CAR-Treg cells and nanoparticle-mediated delivery to the CNS. Third, host factors such as aging, diabetes, and hypertension impair Treg stability and function, suggesting that personalized immunomodulatory strategies may be required.

Future research should move beyond descriptive characterization of Treg responses toward the development of precision immunotherapeutic strategies for ICH. Single-cell transcriptomics and spatial transcriptomics will enable comprehensive characterization of Treg heterogeneity, activation states, and dynamic interactions with microglia, astrocytes, endothelial cells, and infiltrating immune cells within the injured brain. Integrating these approaches with longitudinal immune profiling may facilitate the identification of predictive biomarkers for patient stratification, treatment timing, and therapeutic responsiveness. In parallel, advances in targeted delivery platforms, including brain-homing nanoparticles, engineered Treg products, and antigen-specific CAR-Treg cells, may improve tissue specificity while minimizing systemic immunosuppression. Ultimately, combining multi-omics technologies with biomarker-guided precision immunotherapy is expected to accelerate the clinical translation of Treg-based interventions and enable personalized treatment strategies for patients with ICH.
